# Understanding Patient Perspectives and Experiences in Severe Hemolytic Disease of the Fetus and Newborn: Insights from Qualitative Interviews

**DOI:** 10.1055/a-2948-6478

**Published:** 2026-09-09

**Authors:** Adele Barlassina, Molly Sherwood, Divya Mohan, Marco Boeri, Ellen Janssen, Alexis Krumme, Laura M. Bozzi

**Affiliations:** 1 OPEN Health HEOR and Market AccessMarlowUnited Kingdom of Great Britain and Northern Ireland; 2 Allo Hope FoundationTuscaloosaAlabamaUnited States; 3 Johnson & JohnsonTitusvilleNew JerseyUnited States; 4 Johnson & JohnsonRaritanNew JerseyUnited States

**Keywords:** hemolytic disease of the fetus and newborn, lived experiences, patient perspectives, patient preference study, qualitative research, interviews

## Abstract

**Objective** Maternal red cell alloimmunization is a rare pregnancy condition that can cause hemolytic disease of the fetus and newborn (HDFN), which can be serious and fatal. As subsequent alloimmunized pregnancies carry HDFN risk, families’ future pregnancy decisions may be affected. Findings from qualitative interviews in alloimmunized mothers are presented to understand experiences and perspectives during their previous pregnancy affected by severe HDFN, aiming to inform patient-centered interventions.

**Study Design** This article presents qualitative findings of a broader mixed-methods study published elsewhere. Semi-structured 1:1 interviews were conducted via Microsoft Teams with adults (≥18 years) who experienced a pregnancy within the past 5 years affected by severe HDFN (i.e., intrauterine transfusion, fetal loss after 14 weeks’ gestation, or neonatal death within 4 weeks of birth due to HDFN). A patient advocacy group ensured study design’s relevance, recruited participants, and helped interpret results. Interviews were audio-recorded, transcribed, and analyzed to identify key themes.

**Results** Of 28 participants, 27 completed interviews. Participants reported a median 2 (range, 1–5) affected HDFN pregnancies and a median 4 (1–11) intrauterine transfusions during their most recent pregnancy affected by severe HDFN. Seven themes were identified: (1) diagnosis communication and information seeking, (2) emotional journey at diagnosis, (3) navigating the treatment regimen, (4) physical/emotional challenges during pregnancy, (5) impacts on social life, (6) experience at delivery, and (7) postdelivery adjustment and reflections. Results indicated that participants felt inadequately informed about their diagnosis, experienced significant emotional distress, along with difficulties managing intense monitoring/treatments. Many described their delivery as traumatic and their postpartum as very stressful, which influenced their decision about additional pregnancies.

**Conclusion** These findings highlight opportunities to strengthen communication and support for pregnant individuals experiencing severe HDFN. Developing comprehensive support models and improving care, guided by patient preferences, could address the needs of affected individuals and families.

**Key Points**

- Severe HDFN pregnancy caused high stress/anxiety.
- HDFN monitoring/invasive care created major burdens.
- HDFN treatment impaired emotional well-being/daily life.
- Improved communication/support is needed for HDFN care.
- Patient-centered guidelines are essential for HDFN care.

## Introduction

Hemolytic disease of the fetus and newborn (HDFN) is a rare disease that can occur when there is a mismatch between the antigens on maternal and fetal red blood cells (RBCs). 1 As a result of this incompatibility, maternal alloantibodies produced by a pregnant person’s immune system cross the placenta and attack fetal RBCs, causing fetal RBC hemolysis. 1 HDFN symptoms range from mild to severe; mild disease may be limited to mild fetal anemia and hyperbilirubinemia requiring neonatal phototherapy and/or top-up transfusion, while severe disease can lead to fetal hydrops and fetal/neonatal mortality. 2 3 The severity of HDFN tends to increase with each subsequent affected pregnancy, with earlier gestational onset of disease posing greater risks to the fetus. 4

While there are no approved pharmacological treatments for HDFN, standard-of-care management involves serial antibody titer assessments, monitoring for fetal anemia by middle cerebral artery (MCA) Doppler ultrasound, cordocentesis confirmation of fetal anemia, and intrauterine transfusion (IUT) of cross-matched, antigen-negative RBCs, which is indicated during pregnancy for severe fetal anemia to avoid hydrops fetalis and fetal loss. 2 However, the IUT procedure is an invasive intervention that can be associated with complications, including an increased risk of infections, emergency cesarean delivery, premature birth, or fetal loss, as well as increased maternal alloimmunization. 5 6 7 8 Intravenous immunoglobulin (IVIG) is also sometimes used off-label for the management of severe HDFN to delay the onset of fetal anemia and postpone the need for early IUT. 9 HDFN may also have serious clinical implications for the affected newborn, including hyperbilirubinemia and anemia, which can progress to kernicterus, cardiac failure, and death if improperly managed, underscoring the importance of early detection and management of this condition. 10 11 12

The emotional burden experienced by pregnant individuals affected by severe HDFN is profound. 13 The treatment journey during a pregnancy affected by severe HDFN considerably impacts a patient’s daily life, with the majority reporting difficulty coordinating care for children at home, missing work for monitoring and treatments, and being unable to engage in daily life. 13 The willingness of individuals at risk of severe HDFN to pursue a known alloimmunized pregnancy can be influenced by both treatment-specific and personal factors, including disease severity, medical history, and safety concerns for both the pregnant individuals and their unborn child.

To understand how patients’ treatment decisions are affected by their experiences, it is important to understand the experiences and most relevant aspects of treatments from individuals who experienced a pregnancy affected by severe HDFN. The findings from a qualitative interview study presented here describe participants’ experiences and perspectives on the treatment journey to understand how the lives of individuals with pregnancies affected by severe HDFN have been impacted. This qualitative research can inform the development of patient-centered interventions tailored to address specific patients’ needs, beliefs, and preferences, as well as facilitate development of hypotheses for further study. 14 To support knowledge translation and broader dissemination of key concepts for non-specialist audiences, a companion infographic summarizing the main clinical messages and findings is provided in **Supplemental Material S1** (available in the online version only).

## Materials and Methods

### Participants

Participants were recruited by a patient advocacy group for alloimmunized individuals and people affected by severe HDFN. Eligible participants were adults who had experienced a pregnancy affected by severe HDFN at ≥18 years of age within the past 5 years prior to the study, with severe HDFN defined as having one of the following criteria: received IUT for the management of HDFN, had a pregnancy loss after 14 weeks of gestation due to HDFN, or had an HDFN-associated neonatal death within the first 4 weeks of birth. To avoid misinterpretations due to language or cultural differences, participant eligibility was restricted to native English speakers from the United States, Canada, or the United Kingdom. Participants who had experienced multiple qualifying pregnancies were asked to refer to their most severely affected pregnancy during the interview. Those who were pregnant at the time of the study were asked to provide answers based on their previous qualifying pregnancy. The qualitative interviews were completed between August and October 2024.

This study was reviewed by Pearl Institutional Review Board, which assessed the submitted documents for exempt research determination, including the informed consent forms, survey instrument, interview guide, and study protocol, and granted an exemption from full institutional review board review.

### Study Design

This study used qualitative interviews to explore various aspects of the experiences of participants affected by severe HDFN. Qualitative research is well-suited to address the objectives of this study as it captures the complexity and diversity of individuals’ experiences, perspectives, and decision-making processes regarding their health and medical treatment. 15 16 This qualitative component was part of a broader mixed methods study aimed at outlining the medical and treatment preferences in participants affected by severe HDFN. Participants were required to complete an online best worst scaling (BWS) survey to identify their priorities in the selection of treatments for severe HDFN. A thresholding technique survey was also conducted to understand acceptable risks levels of treatments for HDFN. The findings of these BWS and thresholding technique analyses are detailed in a separate publication. 17 Insights from the BWS survey were incorporated into the qualitative interviews, and the information derived from these interviews was used to gain a deeper understanding of the ranking priorities for severe HDFN treatments identified in the BWS survey.

Given the rarity of the condition, this mixed-method approach allowed comprehensive data extraction from a limited sample size. The study was conducted in collaboration with members of a patient advocacy group actively involved in the study team and provided contributions to study design, participant recruitment, and interpretation of results.

Semi-structured 1:1 qualitative interviews, of approximately 1 hour each, were conducted virtually via Microsoft Teams to understand the experiences of individuals affected by severe HDFN. The interview guide covered a range of topics including: the experience of an HDFN diagnosis during pregnancy; how the condition was monitored; experiences during delivery; and emotional experience throughout the postpartum period.

### Data Analyses

Descriptive statistics were used to summarize participants’ sociodemographic and clinical characteristics related to their severely affected HDFN pregnancy. Interviews were audio-recorded, professionally transcribed, and de-identified. All de-identified transcripts were uploaded to ATLAS.ti for data management, coding, and analysis, and were accessible only to members of the study team. Interview transcripts were analyzed using thematic analysis, a method for identifying patterns in data to provide a wider interpretation of the data, adapted from guidance by Braun and Clarke. 18

The thematic analysis was executed in accordance with the following process: (1) familiarization with the data; (2) generating and applying the code book; (3) searching for themes; (4) reviewing themes; and (5) defining and naming themes. Specifically, two analysts independently familiarized themselves with four transcripts. An initial code book was then developed using a hybrid deductive/inductive approach, 19 combining pre-identified concepts in the interview guide and new concepts from the familiarization step. To ensure coding reliability, the analysts independently coded the same two transcripts before meeting to discuss any required edits to the code book (including new codes identified). Inter-rater agreement was assessed in ATLAS.ti using the Kappa ( *K* ) coefficient, with agreement of 0.81 indicating substantial agreement between analysts. The code book was then applied to all transcripts, with regular meetings between analysts to discuss any update required to the code book and resolve any discrepancy in its application. Once all transcripts were coded, data were reviewed to search for patterns and relationships between codes, forming overarching themes.

## Results

### Participants

A total of 28 participants were recruited and completed the initial survey; 27 participants completed the qualitative interviews. Sociodemographic and clinical characteristics related to the most severely affected HDFN pregnancy are presented in Table 1 . Of the 28 participants, the median age was 35 years (range, 26–40), 93% lived in the United States, and 14% were pregnant at the time of taking the survey. A total of 75% of participants had completed a college or advanced degree, 75% were employed full-time, and 32% reported a change in employment status due to severe HDFN.

**Table 1:** Sociodemographic and clinical characteristics related to the most severely affected HDFN pregnancy.

Characteristic	Participants ( *N* = 28)
Age, median (range), years	35 (26–40)
Currently pregnant at the time of interviews, *n* (%)	4 (14.3)
Location, *n* (%)	
United States	26 (92.9)
Canada	2 (7.1)
Distance from treatment center, *n* (%)	
<10 miles	4 (14.3)
10–49 miles	14 (50)
50–99 miles	3 (10.7)
100–500 miles	7 (25)
Number of visits to treatment center, *n* (%)	
1–5	5 (17.9)
6–10	1 (3.6)
11–20	2 (7.1)
21–50	12 (42.9)
>50	8 (28.6)
Relocation for treatment, *n* (%)	
Yes	2 (7.1)
No	26 (92.9)
Number of IUTs received in most severely affected HDFN pregnancy, median (range) ^a^	4 (1–11)
Family size, median (range) ^a^	4 (3–8)
Number of affected HDFN pregnancies, median (range) ^a^	2 (1–5)
Level of education, *n* (%)	
Some college or technical school	7 (25.0)
College graduate	9 (32.1)
Advanced or graduate/postgraduate degree	12 (42.9)
Employment status, *n* (%)	
Student	1 (3.6)
Employed	21 (75.0)
Self-employed	1 (3.6)
Homemaker	8 (28.6)
Change in employment status due to HDFN, *n* (%)	
Yes	9 (32.1)
No	18 (64.3)
Prefer not to answer	1 (3.6)

Abbreviations: HDFN, hemolytic disease of the fetus and newborn; IUT, intrauterine infusion.

^a^ From qualitative interviews ( *N* = 22–26).

Among the participants who completed the qualitative interviews, the median family size was four living members, including parents (range, 3–8). The median number of affected pregnancies was two (range, 1–5), and the median number of IUTs received during their most severely affected pregnancy was four (range, 1–11).

### Qualitative Interviews

Seven themes emerged from participants’ descriptions of their experiences and perceptions ( **Supplemental Fig. S1** , available in the online version only), as described below. Percentages are used to indicate the proportion of participants who raised or discussed a given theme during interviews. As interviews were semi-structured, the absence of explicit mention does not imply absence of experience, and percentages should not be interpreted as prevalence estimates.

#### Theme 1: Diagnosis Communication and Information Seeking

Participants received varying levels of explanation from their health care providers (HCPs) about the implications of an HDFN diagnosis for their pregnancy. Overall, 67% of participants raised concerns about having received insufficient or unclear information regarding their diagnosis and its implications for their pregnancy, leaving many of their initial doubts and concerns unaddressed ( Table 2 ). All participants reported seeking supplementary information from sources outside their HCPs to deepen their understanding of the diagnosis (i.e., internet, podcasts, social media, patient advocacy groups). Participants (52%) emphasized the positive role that patient advocacy groups and social media communities played in helping them understand their condition.

**Table 2:** Patient experiences and perspectives during HDFN diagnosis and treatment.

Diagnosis communication and information seeking	
Inadequate information received from HCPs	*“At that point, they [OBGY] didn’t discuss too much about it with me. I don’t think she knew very much about it. She just was telling me that this was an issue and that it could potentially be an issue in subsequent pregnancies.” P10* *“They didn’t tell me much about it at all. They didn’t really tell me anything about it. I pretty much did my own research on it.” P07*
Seeking external sources of information	*“It was actually my mom who had found [name]’s group, the Allo Hope Foundation, which truly was the only source of resources that I could find that was relevant enough to give me an abundance of information, which helped us really kick off this process and ensure that we did get the care needed.” P13*
**Emotional journey at diagnosis**	
Emotional distress	*“I was extremely anxious, upset, sad, terrified, disappointed, confused.” P09*
Emotional despair	*“It felt pretty devastating because we wanted a few more kids, and then it became a huge stressor that now I’m high risk, and would that be a selfish decision of me to have more kids, knowing that my babies might be affected?” P23*
Cognitive strain	*“Yes, actually, I had a really tough time mentally. Physically, I was fine. I didn’t have any physical pain or anything like that. I think that was probably the more frustrating part because outwardly, I looked fine, I looked normal. It looked like a normal pregnancy, but mentally, I really struggled with the diagnosis because it was really hard for me to understand that my body was doing this to my unborn child.”* P05
Concerns	*“When I learned that my antibodies would affect any future pregnancy I had, I was concerned about what a pregnancy could look like. Would the baby survive, what kind of treatments would I need, what was that like, etc.?” P24*
**Navigating the treatment regimen**	
Coordinating care/reliance on support systems	*“Just because I already had two children at home, so my husband would stay with them, and I had to leave work to be able to make all the appointments, and just coordinating everything was difficult.” P10*
Advocating for care	*“I feel like I was focused on being the advocate for myself. I didn’t enjoy any of the pregnancy.” P20*
Negative experiences with HCPs during monitoring	*“Yes, they [OBGY] were my main point of contact, but they refused to have any part of my diagnosis. They said that it was up to MFM to care for that.” P23*
Impact on daily life	*“Yes, again, just major work challenges; usually needed the day of the procedure and the day after the procedure off from work, and then sometimes the day before the procedure. I also had to go in for bloodwork, and so, it just impacted my work schedule. Like I said, I took that intermittent leave. Yes, I very thankfully didn’t have other kids at that time. Otherwise, that would have been a nightmare. And trying to manage them and all that. Mostly work, logistic type stuff.”* P01

Abbreviations: HCP, health care provider; HDFN, hemolytic disease of the fetus and newborn; MFM, maternal–fetal medicine; OBGY, obstetrics and gynecology.

#### Theme 2: Emotional Journey at Diagnosis

Participants (56%) reported emotional distress, including feeling fearful/scared, anxious, worried, stressed, and upset, when they first learned of alloimmunization and HDFN–associated risks to the fetus/neonate ( Table 2 ). Participants (30%) reported emotional despair, feeling devastated, hopeless, heartbroken, overwhelmed, and lonely. Other participants (30%) highlighted the cognitive strain of trying to make sense of the diagnosis and the treatment required, which caused confusion and frustration, as well as difficulty accepting the diagnosis. Some specific concerns were also raised, mainly related to the health of their developing fetus/neonate and potential impact on subsequent pregnancies.

#### Theme 3: Navigating the Treatment Regimen

Participants (26%) highlighted the burden of coordinating their care throughout their pregnancy, reporting difficulties with scheduling medical appointments, planning childcare, managing work responsibilities, and keeping track of test results ( Table 2 ). Participants (48%) also felt the need to advocate for their care, including asking for medical tests and procedures, pressing for frequent monitoring, seeking monitoring from experts, and requesting family support during medical procedures. While navigating treatment, participants (33%) reported positive experiences with their care team, including a clear understanding of medical procedures, sensitive and responsive care, and feeling heard. Those reporting negative experiences (33%) described a lack of involvement from their primary HCPs and over-reliance on specialists, a lack of trust, perceived insensitive treatment, vulnerability during medical procedures, and long hospital waiting times.

Participants also highlighted the difficulties of managing the intense monitoring and treatment regimens and the impacts on daily life. These included diminished ability to work (44%) and indirect costs associated with medical appointments (52%; e.g., childcare for other children, parking, and gas). Participants (56%) also reflected on the impact of the frequent medical appointments and the associated stress on their families, noting that they had less time to spend with their family members and that their families were impacted by the emotional strain of a pregnancy affected by severe HDFN. Some (33%) stressed the importance of their support systems (e.g., partner, extended family, etc.) to manage daily tasks, especially childcare.

#### Theme 4: Physical and Emotional Challenges during Pregnancy

When describing their experiences with IUTs, some participants highlighted the emotional impacts of the IUTs, such as feeling scared (37%), anxious (26%), stressed (33%), sad (11%), and helpless (7%; Table 3 ). Both IUTs and IVIGs were associated with physical symptoms. These included feeling pain, tiredness, and bruising/soreness during IUT treatments; and migraine/headaches, tiredness/exhaustion, and weakened/collapsed veins during IVIG treatment. Practical challenges during the IUTs were also reported, such as difficulties with needle insertion, needle phobia, and the need to stay still during procedures.

**Table 3:** Patient experiences during a severely affected HDFN pregnancy and its impacts on social life.

Physical and emotional challenges during pregnancy	
Negative emotions and physical impacts associated with IUTs	*“I was scared, and then I was sad. I was sad that my son had to go through this, because I knew, at that point, once they said we had to start doing these, I knew he was really sick.” P10* *“So, during the IUT, obviously, getting all the needles and IV or whatever is just painful. The actual IUT is a little bit painful, not awful, but you can definitely feel the needle going in, and then the day after, I would be really sore.” P02*
Negative emotions and physical impacts associated with IVIGs	*“And so, there was that that I had to do, and then the IVs never went well. A couple of times it took them three tries to get the IV in right. So, that was just never fun. So, it was more after the first three, and he was fine. I felt confident that he was going to make it through this. So, I wasn’t necessarily fearful that he was going to die. It was more just, not super-excited about just going through the process.” P02* *“And I had a lot of side-effects from them. I was very fatigued. I had a lot of migraines. I did not feel well. I felt kind of sick.” P03*
**Impacts on social life**	
Impacts on family relationships	*“On the average day, especially an IUT week, we’re at maybe 50% capacity, if not lower than that, on just basic being able to do dishes, maybe cook a meal, help take care of our daughter. So, that was definitely hard as well. It was just not feeling like I could give my husband an equal partner.” P02* *“I mean, it took a lot of time away from my son, he was only two at the time. It’s hard to explain to a 2-year-old why you’re constantly gone and why you’re going to appointments and that kind of thing.” P26*
Challenges in social interactions	*“And many friends and family didn’t get it, and they tried their best. But, really, the majority of the peer support I received was from the foundation.” P01*

Abbreviations: HDFN, hemolytic disease of the fetus and newborn; IUT, intrauterine transfusion; IV, intravenous; IVIG, intravenous immunoglobulin.

#### Theme 5: Impacts on Social Life

Participants (56%) reported a significant impact on family relationships, including with partners and other children ( Table 3 ). Two participants reported the difficulty of sharing the experience of a pregnancy affected by severe HDFN with extended family and friends; one participant described withdrawing from social life due to the intense focus on the pregnancy, while another reported feeling misunderstood and isolated, despite receiving offers of support, because loved ones were unable to fully comprehend the nature of their experience.

#### Theme 6: Experience at Delivery

More than half of participants (67%) discussed delivering their babies before the due date, between 27 and 38 weeks’ gestation, either through induction of labor or by cesarean delivery ( Table 4 ). Changing the birth plan from a natural birth to an induction of labor consistent with treatment guidelines was described as a negative experience by some participants (41%) who had envisioned their delivery in a certain way but had to adapt to a different outcome. Three participants reported undergoing emergency deliveries due to complications that arose during medical procedures. Positive experiences during delivery were described by few respondents (15%) and included feeling well-informed and prepared for the process, as well as experiencing effective communication and coordination between care teams (i.e., between the primary care team and staff from the neonatal intensive care unit [NICU]). Few participants (7%) described negative experiences during delivery, which were related to a perceived lack of coordination between the primary care team and the pediatric team. This lack of coordination led to uncertainty about whether the newborn would receive appropriate care, as well as frustration over limited communication from the pediatric team and difficulty recalling interactions with the HCPs following birth. Emergency deliveries were also described as scary and traumatic.

**Table 4:** Patient experiences and perspectives during delivery and postdelivery of severely affected HDFN newborns.

Experience at delivery	
Early delivery and change in birth plan	*“So, I had my second IUT at 32 weeks, and my baby’s heart rate dropped during that procedure, so they made the decision to perform an emergency cesarean, and my son was delivered by cesarean at 32 weeks.” P24* *“It was very scary, and it was very traumatic.” P14*
Positive experience with HCPs preparing for delivery	*“Ahead of time, leading up to it, I had a NICU tour and a discussion with the neonatologist that would be caring for my son, and so my MFM arranged that, which I appreciate. My MFM made sure that we had matching donor blood on-hand before the delivery, in case I needed a blood transfusion or my son needed an urgent blood transfusion. So, that was helpful, and I felt, I think, as prepared as possible for that.” P11*
Negative experience with HCPs at delivery	*“And then when I delivered my little guy and the pediatric team was on call there and took him, I don’t even know what they did. Like there wasn’t… I just don’t think whether I was in the mindset. I literally can’t remember if anyone even spoke to me. They just took him, did whatever they needed to do, and I felt like that was it. That’s all I saw of them in the delivery process. It felt a little, I don’t know, just a thorough disconnect there in terms of what we prepare the care that they need is, from our support group and getting each mom ready together to be like, just advocate for this. Make sure they’re doing this.” P13*
Postpartum challenges	*“That, honestly, was so much harder than the pregnancy itself. I think during the pregnancy, there’s a lot more fear just because you can’t see him, hold him, know what the needs are, but most of the things that caused pain and discomfort were done to me and not him. So, I think watching him go through some of those procedures and lab draws and appointments, it was just really hard as a new mom.” P25*
**Postdelivery adjustment and reflections**	
Impact on emotions	*“But, yes, I think PTSD is a perfect term for what I have, because there are definitely moments when I hear about premature babies or something, and I just get very almost emotional, and the anxiety comes roaring back, just remembering. Even driving down to Santa Barbara towards the hospital, because it was the same route every day, I even struggle sometimes driving down to that general area because of what we went through.” P05*
Impact on parenting role	*“I think it just makes me try to... Once they were born, it’s like I really soaked in everything with them because I didn’t really get to enjoy my pregnancies.” P22*
Impact on future pregnancy decisions	*“So, I decided, because statistically, generally, it gets worse each consecutive pregnancy if the baby is affected, so I decided that was it for me. I was not going to do this again. So, I got my tubes out during the C-section, mostly because of this.” P02*

Abbreviations: HCP, health care provider; HDFN, hemolytic disease of the fetus and newborn; IUT, intrauterine transfusion; MFM, maternal–fetal medicine; NICU, neonatal intensive care unit; PTSD, posttraumatic stress disorder.

Participants (70%) described their experience of having their newborn in NICU after delivery. While most described it as acceptable in light of its necessity for their baby’s health, others (7%) referred to it as the hardest part of the pregnancy, as it prevented them from being physically with their babies for days after birth. A participant mentioned that the hardest part of having their newborn in NICU was not being the one making decisions for their baby.

The most common neonatal health complications that were reported by participants were jaundice (59%) and anemia (52%). Participants also described a range of challenges during the postpartum period as they sought to balance their newborn’s health needs with their own recovery, which included realizing the impact severe HDFN had on their newborn and being hypervigilant of their newborn’s health.

#### Theme 7: Postdelivery Adjustment and Reflections

Some participants reflected on the impact that experiencing a pregnancy affected by severe HDFN had on themselves. Some reported suffering from long-term anxiety (26%) and trauma (70%). The trauma manifested for participants in different ways, from mistrust in doctors to fears of hospitals or certain medical procedures. One participant also reported postpartum depression and another the experience of mourning the loss of a normal pregnancy.

Participants (81%) explained that experiencing a pregnancy affected by severe HDFN had enduring impacts on their parenting role. The fear of losing the child during pregnancy heightened their appreciation for moments spent with their child after birth. About one-third of participants (33%) reported that the experience of their severely affected HDFN pregnancy led them to decide against pursuing additional pregnancies. However, other participants did have subsequent pregnancies and four were pregnant at the time of the study.

## Discussion

These qualitative interviews confirmed that individuals affected by severe HDFN during pregnancy faced significant emotional, physical, and practical challenges related to the diagnosis and management of the disease. Participants in this study felt inadequately informed about their diagnosis, prompting them to seek information independently and advocate for themselves, which led to significant stress and anxiety. Difficulties in managing intense monitoring and treatment regimens, often requiring family support, further emphasized the burden of severe HDFN on daily life, including impact on family relationships and social interactions. Traumatic experiences during delivery and the stressful postpartum period influenced participants’ decisions about future pregnancies, highlighting the profound impact of severe HDFN on long-term family planning and mental health.

These findings align with existing literature on the importance of patient-centered care and the need for tailored approaches for managing these high-risk pregnancies. 3 20 21 22 The recent emergence of multidisciplinary clinical practice guidelines, involving clinicians, patient representatives, and stakeholder organizations, 23 24 could enhance care and support for affected families. These guidelines cover emergency transfusion protocols, follow-up alloimmunization screening, pregnancy monitoring and interventions (e.g., IVIG administration, surveillance of MCA peak systolic velocity), and postnatal management, 23 24 aligning closely with the communication, coordination, and support needs identified in this study. Incorporating patient preferences (e.g., providing more comprehensive communication during diagnosis and resources throughout pregnancy, treatment, and postpartum) into treatment planning, in collaboration with HCPs and patient advocacy groups, may strengthen the overall support for individuals with severe HDFN during their pregnancies, which may contribute to maternal well-being, patient satisfaction, adherence to treatment, and fetal/neonatal health outcomes.

The findings also highlighted opportunities for improved communication and support to address the significant emotional and practical challenges of individuals with pregnancies affected by severe HDFN. This need is not limited to severe cases; recent comparative research on severe and non-severe HDFN pregnancies found that mental health support was offered to 23% of participants during pregnancies affected by severe HDFN ( *n* = 22) and to 25% during pregnancies to those affected by non-severe HDFN ( *n* = 105), 13 underscoring a critical gap in care that spans the full spectrum of disease severity. Similarly, many of the participants in this study expressed the value of wraparound support from patient advocacy groups and social media communities as a source for education, expert referrals, and empowerment for informed decision-making. Participants also reflected on difficulties during delivery and the postpartum period, underscoring the need for stronger support from HCPs, as well as structured follow-up to address both physical and psychological recovery.

The strengths of this study include the collaboration with a patient advocacy group, which helped to optimize the study protocol, survey questions, and participant recruitment, leveraging their patient-centered insights into the disease. The patient advocacy group also conducted training for the study personnel, given the sensitive nature of patient interviews discussing high-risk pregnancies. Furthermore, the recruitment for this study was not center-specific, allowing for varied sources of treatment experience without relying on a single high-volume HDFN referral center.

Potential limitations of this study include the small sample size due to the rarity of the condition and limited geographic diversity among participants. These factors may restrict the generalizability of the findings to individuals in other regions of the world. Additionally, there is a potential selection bias due to recruitment of participants through a patient advocacy group. Patients involved with a patient advocacy group might tend to seek more information and be more informed about their condition. 25 Additionally, they may have been more proactive in seeking supplementary information and be more closely connected to advocacy-related resources and networks than the broader patient population.

Further, some patients were pregnant at the time of the study, and there may be other unmeasured confounding variables, such as demographic, socioeconomic status, and educational background, that could influence the results. In addition, data on the time since the eligible pregnancy was not provided, and there is a potential for recall bias. Future research should include more representative samples of patients with diverse cultural backgrounds and medical literacy to support the findings and to provide a comprehensive understanding of the experiences and perspectives of patients with pregnancies affected by severe HDFN globally.

## Conclusion

This qualitative interview study highlights the emotional, physical, and practical challenges reported by individuals with pregnancies affected by severe HDFN. Participants emphasized the value of patient advocacy groups and social media communities as a source of educational information, aiding in their understanding of severe HDFN and supporting their informed decision-making. There are opportunities for improved communication between patients and HCPs on the condition and treatment options. Additional support for individuals with pregnancies affected by severe HDFN is needed and can be made available by integrating patient preferences and utilizing patient advocacy groups.

## Supplementary Material

Ergänzendes Material Supplementary Material
