# Supplementary material for: Understanding Patient Perspectives and Experiences in Severe Hemolytic Disease of the Fetus and Newborn: Insights from Qualitative Interviews
Source: AJP Rep. 2026 Sep 9;16(3):e171–8. doi: 10.1055/a-2948-6478 (PMC13557808; doi:10.1055/a-2948-6478)
Supplement: Supplementary file 1 [file 10-1055-a-2948-6478_29504595.pdf]

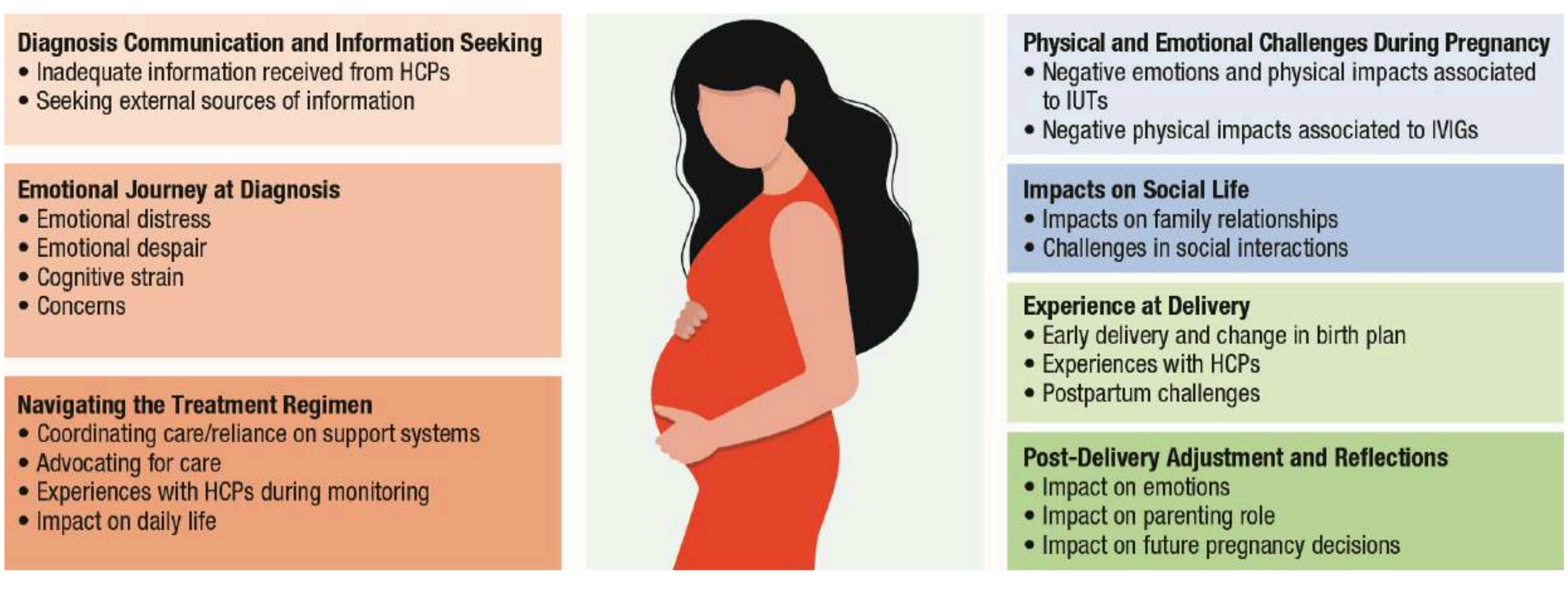
Diagnosis Communication and Information Seeking
• Inadequate information received from HCPs
• Seeking external sources of information
Emotional Journey at Diagnosis
• Emotional distress
• Emotional despair
• Cognitive strain
• Concerns
Navigating the Treatment Regimen
• Coordinating care/reliance on support systems
• Advocating for care
• Experiences with HCPs during monitoring
• Impact on daily life
Physical and Emotional Challenges During Pregnancy
• Negative emotions and physical impacts associated to IUTs
• Negative physical impacts associated to IVIGs
Impacts on Social Life
• Impacts on family relationships
• Challenges in social interactions
Experience at Delivery
• Early delivery and change in birth plan
• Experiences with HCPs
• Postpartum challenges
Post-Delivery Adjustment and Reflections
• Impact on emotions
• Impact on parenting role
• Impact on future pregnancy decisions

Thieme

# Understanding Patient Perspectives and Experiences in Severe Hemolytic Disease of the Fetus and Newborn: Insights From Patient Interviews

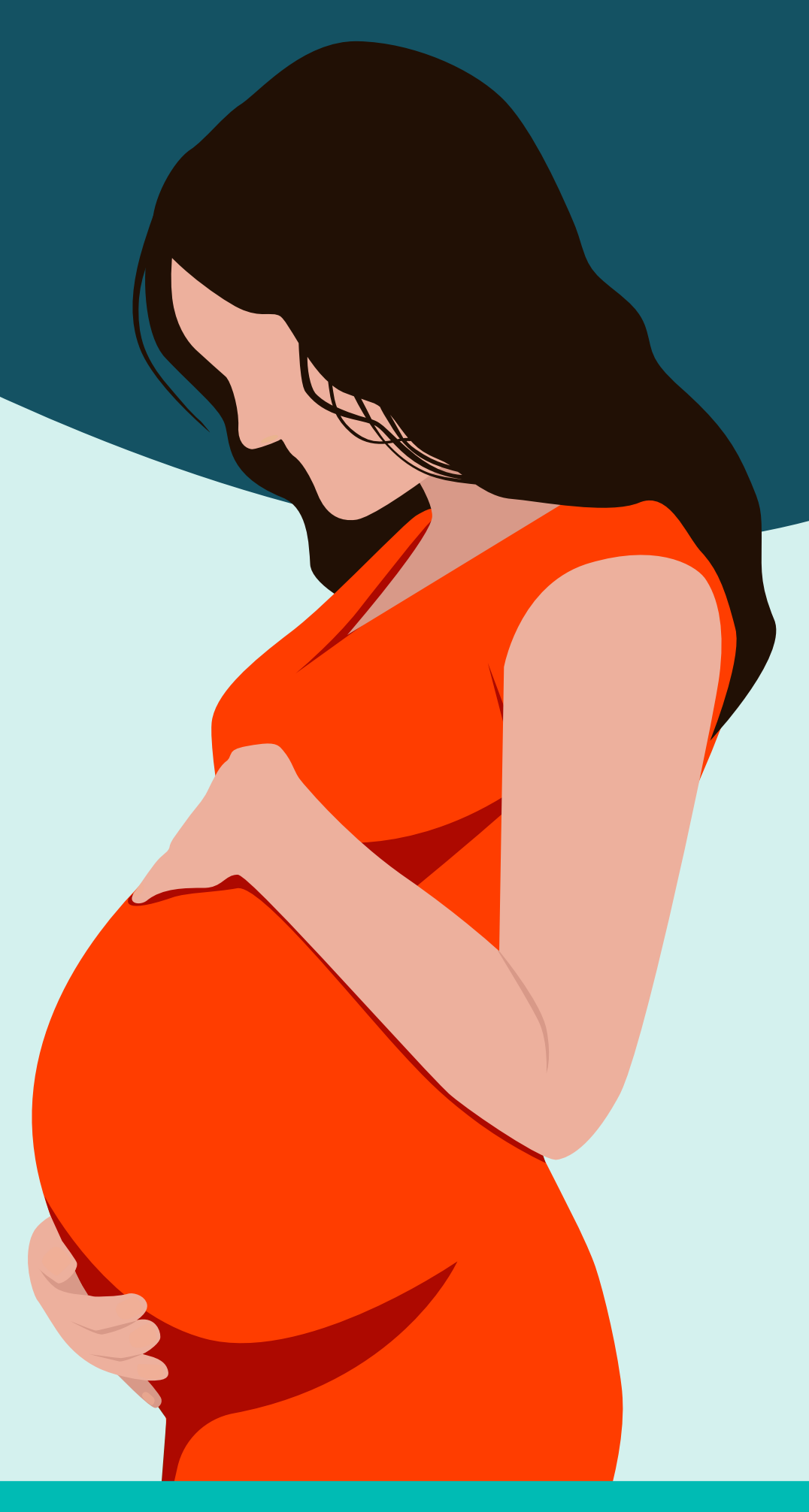

## Key Takeaways

- **Pregnant individuals affected by severe hemolytic disease of the fetus and newborn (HDFN) face substantial emotional, physical, and practical challenges** throughout the phases of diagnosis, treatment management, childbirth, and postpartum that significantly affect their well-being and daily life.
- Results underscore **opportunities to strengthen communication and enhance support** for pregnant individuals experiencing severe HDFN by integrating patient preferences throughout the treatment journey.
- **Developing comprehensive support models and improving care, guided by patient preferences**, could address the needs of affected individuals and families.

## What is HDFN, and how is it diagnosed and treated?

- HDFN is a rare disease caused **by a mismatch between the antigens** (a substance that triggers the body to produce an immune response) **on the red blood cells (RBCs) of a pregnant person and their developing fetus**.

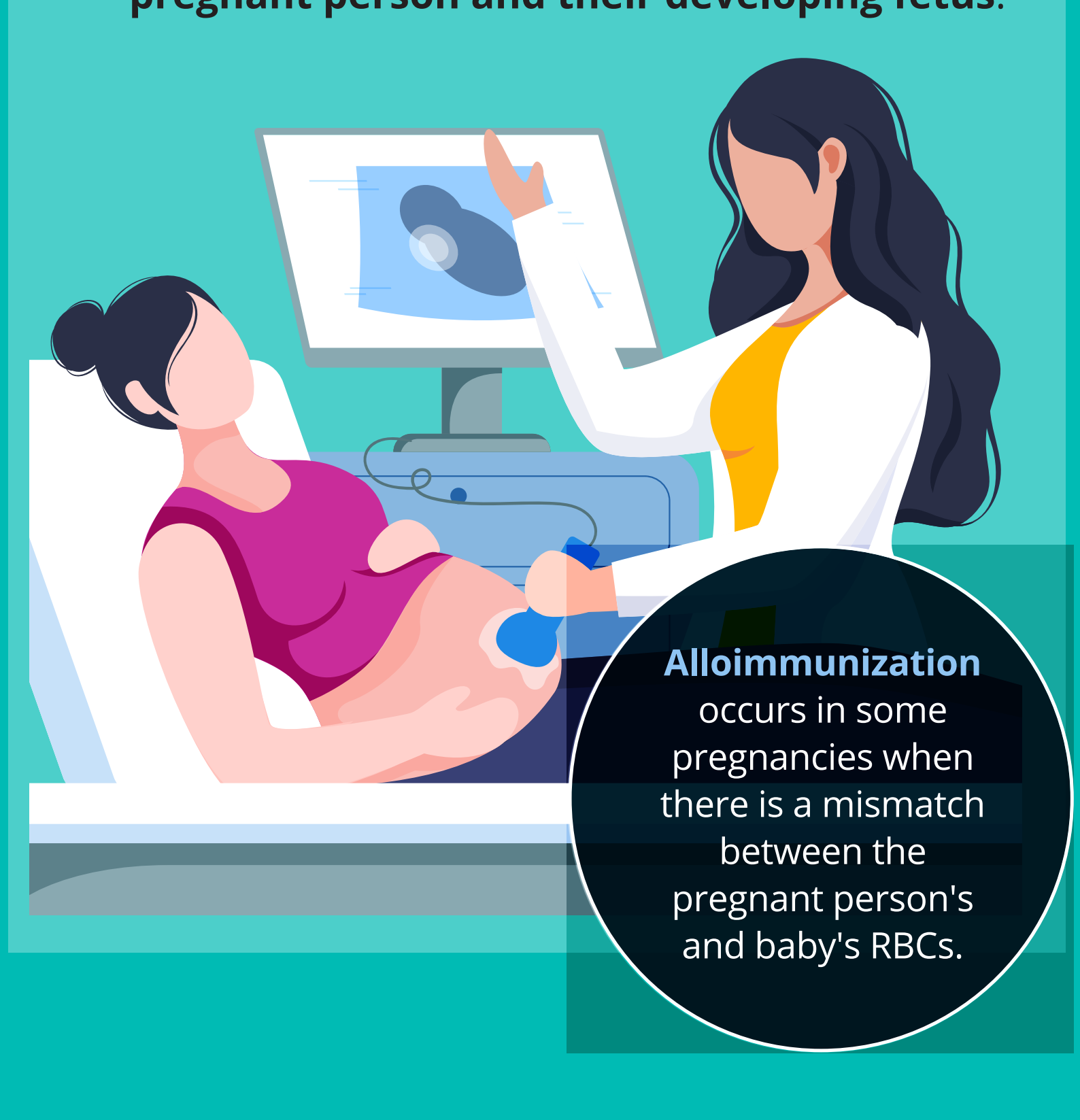

**Alloimmunization** occurs in some pregnancies when there is a mismatch between the pregnant person's and baby's RBCs.

- As a result of this mismatch, the pregnant person's **immune system produces pathogenic antibodies (also called alloantibodies) that cross the placenta and attack the fetal RBCs** to potentially cause this life-threatening condition.
- If untreated, **HDFN can lead to serious consequences for the baby**, such as fetal or neonatal anemia.
- The **severity of HDFN tends to increase with each affected pregnancy**, with the fetus developing anemia earlier in each subsequent pregnancy. **Close monitoring and timely treatment is crucial** to ensure babies affected by HDFN will not have lasting health effects.
- **The current standard of care for fetal anemia** is to monitor the developing fetus using middle cerebral arterial (MCA) Doppler ultrasound; if fetal anemia is present, an intrauterine transfusion (IUT) is administered.
- **An IUT is an invasive procedure** that is associated with complications, including an increased risk of preterm birth or fetal loss.
- **In an IUT, a needle is inserted through the maternal belly and uterus into the baby's umbilical cord or belly** to deliver RBCs that will not be destroyed by the pregnant person's immune system.
- **An IUT of RBCs** is used for severe cases of HDFN.

Given the significant emotional burden of HDFN, the **patient's treatment journey can play a pivotal role** in shaping decisions about pursuing future alloimmunized pregnancies.

## What was the objective of this study?

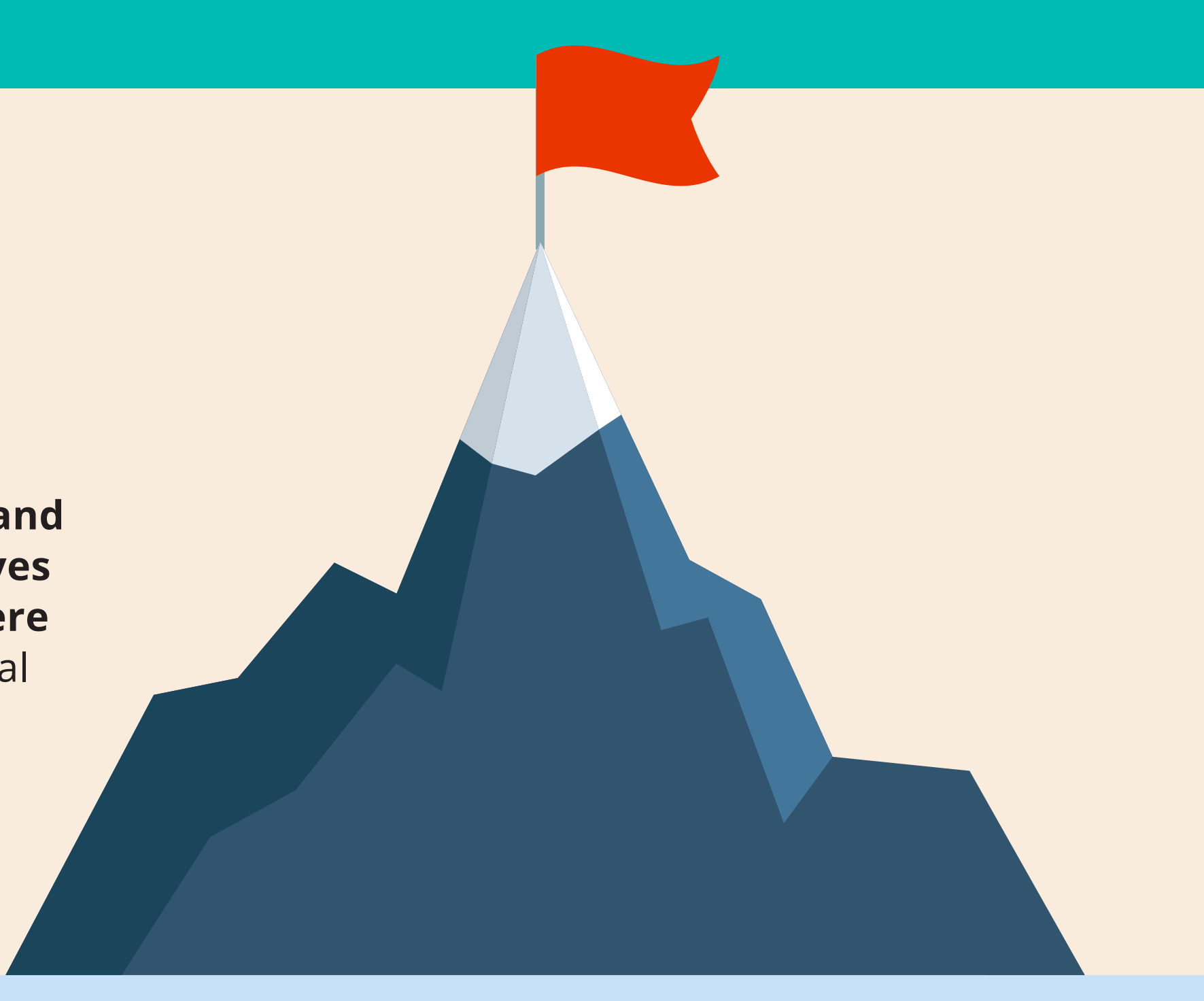

- This study used interviews **to understand the lived experiences and perspectives of individuals whose pregnancies were affected by severe HDFN**, with the goal of informing the development of patient-centered interventions and support strategies.

## Methods

### How were data on pregnancies affected by severe HDFN gathered and analyzed in this study?

In this study, participants who had experienced a pregnancy affected by severe HDFN were interviewed to share their experiences and perspectives on that pregnancy.

- This study was conducted **in collaboration with a patient advocacy group** for families with pregnancies affected by HDFN.
- Data came from **semistructured 1:1 virtual interviews** with the participants (approximately 1 hour).
- The interview guide covered a range of topics, including the **experiences of receiving an HDFN diagnosis**, undergoing **monitoring** during an HDFN-affected pregnancy, the **birth experience**, and dealing with the **emotional impact**.
- Data from the interviews were analyzed using an iterative **thematic analysis**.

**Study population**

Adults (≥18 years of age) with a pregnancy affected by severe HDFN that occurred at ≥18 years of age and within 5 years before the study, with severe HDFN defined as having 1 of the following:

- Received an IUT for the treatment of HDFN
- Pregnancy loss after 14 weeks of gestation due to HDFN
- Had an HDFN-associated neonatal death within the first 4 weeks of birth

Participants resided in the United States or Canada.

## Results

### Sociodemographic and clinical characteristics of the participants related to their most recent severe HDFN pregnancy

- A total of **28 participants** were recruited and completed the initial survey
- Of those surveyed, **27 participants** completed the interviews
- **93%** lived in the **United States**
- The median family size (including parents) was **4 members (range, 3-8)**
- The median age was **35 years (range, 26-40)**
- The median number of HDFN-affected pregnancies was **2 (range, 1-5)**
- **4 participants were pregnant** at the time of taking the survey
- The median number of IUTs received during the participants' most recent pregnancy affected by severe HDFN was **4 (range, 1-11)**

### Seven major themes emerged that explored patient experiences and perspectives during a pregnancy affected by severe HDFN

**1 Diagnosis communication and information seeking**
- Most participants felt **inadequately informed about their diagnosis**.
- All participants **sought external sources of information** to improve their understanding of alloimmunization and HDFN.

**2 Emotional journey at diagnosis**
- Participants experienced significant **emotional distress, despair, and difficulties in understanding the diagnosis**.

**3 Navigating the treatment regimen**
- Participants found the **coordination of their care to be challenging**.
- Participants felt the need to advocate for their care.
- Participants reported **both positive and negative experiences with health care providers (HCPs)** during monitoring and treatment.
- Participants highlighted the **impact on daily life** caused by managing intense monitoring and treatment regimens.

**4 Physical and emotional challenges during pregnancy**
- Participants reported the **negative physical impacts associated with IVIG**.
- Participants reported the **negative emotions and physical impacts associated with IUTs**.

**5 Impact on social life**
- Participants reported a significant **impact on family relationships**.
- Participants faced **challenges in sharing their experiences with others**, which led to social isolation.

**6 Experience at delivery**
- Participants described the challenges related to their **early delivery**, **change in birth plan**, **experience with HCPs**, and **postpartum period**.

**7 Postdelivery adjustment and reflections**
- Participants experienced enduring **anxiety, trauma, postpartum depression**, and **significant impacts on their parenting roles** following delivery.
- Participants' experiences with HDFN **influenced their decision to have more children**.
